# Supplementary material for: Factors that mediate the relationships between household socio-economic status and childhood Attention Deficit Hyperactivity Disorder (ADHD) in children and adolescents: A systematic review
Source: PLoS One. 2022 Mar 1;17(3):e0262988. doi: 10.1371/journal.pone.0262988 (PMC8887716; doi:10.1371/journal.pone.0262988)
Supplement: S1 Table — (DOCX) [file pone.0262988.s001.docx]

**Supporting Information Table S1 Risk of bias (quality) assessment**

|  | Boe et al. (2018) [29] | Foulon et al. (2015) [23] | Meunier et al. (2013) [24] | Miller et al. (2016) [27] | Nguyen et al. (2019) [28] | Russell A. E et al. (2015) [12] | Russell G et al. (2014) [25] | Schmiedeler et al. (2014) [26] |
| --- | --- | --- | --- | --- | --- | --- | --- | --- |
| Criteria |  |  |  |  |  |  |  |  |
| Study design | Cross-sectional | Cohort | Cohort | Case-control | Cross-sectional | Cohort | Cohort | Cohort |
| 1. Was the research question or objective in this paper clearly stated? | Yes | Yes | Yes | Yes | Yes | Yes | Yes | Yes |
| \|  \| \| --- \| \| 2. Was the study population clearly specified and defined? \| | Yes | Yes | Yes | Yes | Yes | Yes | Yes | Yes |
| 3. Was the study population representative of the whole target population? | Yes | X | X | X | Yes | Yes | Yes | X |
| 4. Was the participation rate of eligible persons at least 50%? | X | Yes | X | Cannot Determine | X | Cannot Determine | Cannot Determine | Cannot Determine |
| 5. Were all the subjects selected or recruited from the same or similar populations (including the same time period)? Were inclusion and exclusion criteria for being in the study prespecified and applied uniformly to all participants? | Yes | Yes | Yes | Yes | Yes | Yes | Yes | Yes |
| 6. Was a sample size justification, power description, or variance and effect estimates provided? | NA | NA | NA | X | NA | NA | NA | NA |
| 7. For the analyses in this paper, were the exposure(s) of interest measured prior to the outcome(s) being measured? | NA | Yes | Yes | NA | NA | Yes | Yes | Yes |
| 8. Was the timeframe sufficient so that one could reasonably expect to see an association between exposure and outcome if it existed? | NA | Yes | Yes | NA | NA | Yes | Yes | Yes |
| 9. For exposures that can vary in amount or level, did the study examine different levels of the exposure as related to the outcome (e.g., categories of exposure, or exposure measured as continuous variable)? | Yes | Yes | Yes | Yes | Yes | X* | Yes | Yes |
| 10. Were the exposure measures (independent variables) clearly defined, valid, reliable, and implemented consistently across all study participants? | No | Yes | Yes | Yes | No | Cannot Determine | Yes | Yes |
| 11. Was the exposure(s) assessed more than once over time? | NA | X | X | X | NA | X | X | X |
| \| 12. Were the outcome measures (dependent variables) clearly defined, valid, reliable, and implemented consistently across all study participants? \|  \| \| --- \| --- \| | Yes | Yes | Yes | No | Yes | Yes | Yes | Yes |
| 13.Were the outcome assessors blinded to the exposure status of participants? | X | X | X | X | X | X | X | X |
| 14. Was loss to follow-up after baseline 20% or less or weighting &/or imputation used to account for loss to follow up? | NA | X | X | NA | NA | X | X** | Yes |
| 15.Were potential baseline covariates/confounders variables measured and adjusted statistically for their impact on the relationship between exposure(s), mediators and outcome(s)? | X | Yes | Yes | Yes | Yes | Yes | X | Yes |
| 16. Was Mediation analysis clearly specified & defined? | Yes | Yes | Yes | Yes | Yes | Yes | Yes | Yes |
| 17. Was choice of mediators clearly specified & justified? | X | Yes | Yes | Yes | Yes | Yes | Yes | Yes |
| 18. Were results of mediation analysis clearly presented allowing direct & indirect effects to be distinguished? | X | Yes | Yes | Yes | Yes | Yes | Yes | Yes |
|  |  |  |  |  |  |  |  |  |
| Overall quality assessment | Poor  **Reasons**  Cross-sectional  Low participation rate  Exposure and mediator likely to be conflated  No adjustment for covariates/  confounders  Perceived SES-(mediator) not validated | Fair  **Reasons**  Likely non- representative sample  High attrition rate (>20%) | Fair  **Reasons**  Non-representative sample  Low participation rate  High attrition rate (>20%) | Poor  **Reasons**  Case control  Non-representative sample  Selection of cases & controls unclear    Non-participation rate not stated | Poor  **Reasons**  Cross-sectional  Low participation rate  Over-controlling for SES in mediation analysis  School engagement  neighbor-hood safety and neighbor-hood amenities not validated | Fair  **Reasons**  Participation rate not disclosed  High attrition rate (>20%)  Exposure not clearly defined | Fair  **Reasons**  Cannot determine participation  High attrition rate (>20%)  No adjustment for covariates/  confounders | Poor  **Reasons**  Non-representative sample  Over controlling for ADHD at baseline as it is almost certainly related to both SES at baseline |
| Risk of Bias | High | Moderate | Moderate | High | High | Moderate | Moderate | High |

** State It was not possible to weight the data in analysis of mediation; however, unweighted regression models are often robust in large datasets (see Wolke et al., 2009 [61]
