# Supplementary material for: Factors that mediate the relationships between household socio-economic status and childhood Attention Deficit Hyperactivity Disorder (ADHD) in children and adolescents: A systematic review
Source: PLoS One. 2022 Mar 1;17(3):e0262988. doi: 10.1371/journal.pone.0262988 (PMC8887716; doi:10.1371/journal.pone.0262988)
Supplement: S1 File — (DOCX) [file pone.0262988.s002.docx]

|  |  |
| --- | --- |

Systematic review of factors that mediate the relationships between parental socio-
economic status and childhood ADHD

*Wolfgang Markham, Nicholas Spencer*

Citation

Wolfgang Markham, Nicholas Spencer. Systematic review of factors that mediate the relationships between parental socio-economic status and childhood ADHD. PROSPERO 2020 CRD42020182832 Available from: <https://www.crd.york.ac.uk/prospero/display_record.php?ID=CRD42020182832>

Review question

What factors mediate the relationship between parental socio-economic status and ADHD in childhood and adolescence?

Searches

MEDLINE, EMBASE, PsycINFO, Web of Science

Search dates: (from 1946 to 06 May 2020)

Restrictions: Published in English

Secondary search of reference lists

Unpublished studies will not be sought

Searches will not be re-run prior to the final analysis

Types of study to be included

Inclusion

We will include only primary studies - cohort, cross-sectional, and case control.
Exclusion

We will not include studies that are based on clinical samples or studies of twins. Reviews that are identified in the search will not be included in the systematic review.

Condition or domain being studied

ADHD either medical diagnosis or high score for hyperactivity/inattention on standard psychometric tests in childhood or adolescence as reported by health care professional, teacher or parent

Participants/population

Inclusion:

Children and adolescents under 19 years of age diagnosed as having ADHD or having scores equal to or over the accepted cut-off for hyperactivity/inattention on standard psychometric tests as reported by health care professional, teacher or parent.

Exclusion:

Adults over 18 years of age

|  |  |
| --- | --- |

Children and adolescents under 19 years of age having high scores on standard psychometric tests for externalising behaviour not disaggregated into hyperactivity/inattention and conduct disorder

Children and adolescents under 19 years of age diagnosed with conduct problems

Intervention(s), exposure(s)

Measures of parental socio-economic status that are based on income, education, neighbourhood disadvantage/advantage and other accepted measures of socio-economic status such as housing tenure and financial hardship

Intervention: Any social or behavioural factor that potentially mediates the relationship between parental socio-economic status and childhood/adolescent ADHD or childhood/adolescent hyperactivity/inattention disorder. We will not include biological factors that are based upon, for example, brain morphology.

Comparator(s)/control

Children and adolescents under 19 years of age who have not been diagnosed as having ADHD or high scores for hyperactivity/inattention on standard psychometric tests

Main outcome(s)

ADHD or high scores for hyperactivity/inattention in standard psychometric tests

* Measures of effect

Mediating pathway beta coefficients

Additional outcome(s)

None

* Measures of effect

None

Data extraction (selection and coding)

Study selection

Two reviewers will independently screen records for inclusion and apply eligibility criteria for selecting studies for inclusion in the systematic review. The reviewers will be blinded to each other’s decisions.

Full text articles will be obtained for papers that pass the initial screening phase. The initial screening phase will be based upon the following questions:

Empirical primary research study (i.e. not secondary research/review/evaluation/audit)?

General population-based sample of children and/or adolescents under the age of 19 years (i.e. not a clinical sample or twin study)?

Exposure: one or more measures of parental socio-economic status

Outcome: ADHD or high score for hyperactivity/inattention on standard psychometric tests (externalising behaviour including conduct problems or conduct disorder alone excluded?)

Include a mediation analysis of factors that potentially mediate the relationship between parental socio­economic status (SES) (exposure) and ADHD or hyperactivity/inattention in childhood/adolescence (outcome)?

Use a recognised method for assessing mediation?

Disagreements between the reviewers will be resolved through discussions.

We will record decisions on an excel spreadsheet.

|  |  |
| --- | --- |

Data extraction

The following data will be extracted from study documents

Author

Year

Country

Study type; population; sample size; Attrition (%)

SES measure/s; child's/adolescent's age at SES measurement

ADHD measure; child's/adolescent's age at ADHD measurement; Prevalence (%)

Mediators studied & method

Direct & Indirect effects of SES and mediators (pathway coefficients)

Study investigators will not be contacted for unreported data or additional details

Two reviewers will independently extract data

Disagreements in judgements between the reviewers will be resolved through discussions

We will record decisions on an excel spreadsheet. A software tool will not be used

Risk of bias (quality) assessment

NIH assessment tool for observational, cohort and cross-sectional studies

<https://www.nhlbi.nih.gov>./health-topics/study-quality-assessment-tools

The NIH assessment tool for observational, cohort and cross-sectional studies is the most appropriate we

have found for the purposes of this review with some modification to take into account specific features of

the review particularly mediation.

We will add the following questions to this assessment tool.

Was the study population representative of the whole target population?

Was mediation analysis clearly specified and defined?

Was choice of mediators clearly specified and justified?

Were results of mediation analyses clearly presented allowing direct and indirect effects to be distinguished?

Independent assessment of the risk of bias of the data/quality assessment will be undertaken blind by two

reviewers on papers included following full paper screen

Level of risk assessment of studies will be considered in the results and discussion sections of the report as

part of the narrative synthesis

Disagreements in judgements between the reviewers will be resolved through discussions

The data will be recorded in an excel spread sheet

Strategy for data synthesis

We will identify mediators explaining all/some of the influence of parental socio-economic on ADHD or hyperactivity/inattention among children/adolescents aged under 19 years.

|  |  |
| --- | --- |

We will synthesise quantitative data without meta-analysis (SWiM) because of anticipated sources of

heterogeneity

Grouping of studies for synthesis

Potential mediating factors will be grouped according to whether they are social or behavioural factors

Describe the standardised metric or transformation method used

Direct pathway coefficients or mediating (indirect) pathway coefficients (unstandardized beta coefficients)

Describe the synthesis methods

Summarising effect estimates in order to identify the range and distribution of observed effects. We will vote

count if possible in terms of how often a significant mediating effect was observed but this will depend on

which mediators are investigated and how often and how the mediators were measured.

Criteria used to prioritise results for summary and synthesis

Prioritisation of results will be based upon risk of bias assessments i.e. studies at low risk of bias will be

prioritised over other studies

Investigation of heterogeneity in reported effects

The investigation will be informal and will involve ordering in tables. The ordering will take into account

methodological considerations (study design), the categorisation of mediating factors (social or behavioural)

and risk of bias assessments

Certainty of evidence

Will be based on risk of bias assessments, study design and any bias in relation to only reporting significant

pathway coefficients in the results sections of included papers.

Data presentation methods

A risk of bias table

A table of mediating pathway coefficients be they significant, non-significant or investigated but not reported

in the paper.

Reporting of results

Will be based upon 'Is there any evidence of effect?' taking into account significant, non-significant and non-

reported

pathway coefficients

Limitations of synthesis

Will be based upon 'Is here any evidence of effect?', any reconfiguration of groups used to structure the synthesis, any incomplete reported effect estimates, and risk of bias assessments.

Analysis of subgroups or subsets

We do not plan to do any ‘subgroup’ analysis or investigate potential effect modifiers

Contact details for further information

Wolfgang Markham

[Wolfgang.Markham@warwick.ac.uk](mailto:Wolfgang.Markham@warwick.ac.uk)

Organisational affiliation of the review

University of Warwick
[warwick.ac.uk](http://warwick.ac.uk)

Review team members and their organisational affiliations

Dr Wolfgang Markham. Warwick Medical School, University of Warwick
Professor Nicholas Spencer. University of Warwick

Type and method of review

Narrative synthesis, Systematic review

Anticipated or actual start date

06 May 2020

Anticipated completion date

|  |  |
| --- | --- |

10 September 2020

Funding sources/sponsors

None

Conflicts of interest
Language

English

Country

England

Stage of review

Review Ongoing

Subject index terms status

Subject indexing assigned by CRD

Subject index terms

Attention Deficit Disorder with Hyperactivity; Economic Status; Humans; Social Class; Socioeconomic Factors

Date of registration in PROSPERO

30 June 2020

Date of first submission

28 April 2020

Stage of review at time of this submission

**Stage Started Completed**

Preliminary searches Yes Yes

Piloting of the study selection process Yes Yes

Formal screening of search results against eligibility criteria No No

Data extraction No No

Risk of bias (quality) assessment No No

Data analysis No No

*The record owner confirms that the information they have supplied for this submission is accurate and complete and they understand that deliberate provision of inaccurate information or omission of data may be construed as scientific misconduct.*

*The record owner confirms that they will update the status of the review when it is completed and will add publication details in due course.*

Versions

30 June 2020

|  |  |
| --- | --- |
