# Supplementary material for: Factors that mediate the relationships between household socio-economic status and childhood Attention Deficit Hyperactivity Disorder (ADHD) in children and adolescents: A systematic review
Source: PLoS One. 2022 Mar 1;17(3):e0262988. doi: 10.1371/journal.pone.0262988 (PMC8887716; doi:10.1371/journal.pone.0262988)
Supplement: S3 File — (DOCX) [file pone.0262988.s004.docx]

**Supporting Information S3 File: Search Strategies for Electronic Databases**

ADHD with mediation,

Search run: 01 May 2020

|  | References retrieved | Deduplicated references |
| --- | --- | --- |
| Medline ALL | 291 | 284 |
| Embase | 372 | 185 |
| PsycInfo | 237 | 82 |
| Web of Science | 230 | 75 |
|  |  |  |
|  | 1130 | 626 |

Database: Ovid MEDLINE(R) ALL <1946 to April 30, 2020>

Search Strategy:

--------------------------------------------------------------------------------

1 exp Attention Deficit Disorder with Hyperactivity/ (28316)

2 adhd.mp. (24992)

3 attention deficit hyperactivity disorder*.mp. (24765)

4 attention deficit-hyperactivity disorder*.mp. (24765)

5 attention deficit disorder* with hyperactivity.mp. (28460)

6 addh.mp. (118)

7 1 or 2 or 3 or 4 or 5 or 6 (38943)

8 socioeconomic.mp. or exp Socioeconomic Factors/ (489674)

9 social class.mp. or exp Social Class/ (45751)

10 exp Poverty/ or poverty.mp. (58659)

11 exp Income/ or income.mp. (167377)

12 economic status.mp. or exp Economic Status/ (13828)

13 educational status.mp. or exp Educational Status/ (54363)

14 exp Parents/ (111059)

15 (parent or parents or mother* or father* or maternal or paternal).mp. [mp=title, abstract, original title, name of substance word, subject heading word, floating sub-heading word, keyword heading word, organism supplementary concept word, protocol supplementary concept word, rare disease supplementary concept word, unique identifier, synonyms] (733247)

16 14 or 15 (733247)

17 13 and 16 (11429)

18 8 or 9 or 10 or 11 or 12 or 17 (570831)

19 7 and 18 (2114)

20 (mediating or mediation or mediator* or mediated).mp. [mp=title, abstract, original title, name of substance word, subject heading word, floating sub-heading word, keyword heading word, organism supplementary concept word, protocol supplementary concept word, rare disease supplementary concept word, unique identifier, synonyms] (1203607)

21 moderat*.mp. (568917)

22 attenuat*.mp. (352853)

23 indirect*.mp. (244916)

24 pathway*.mp. (1193193)

25 path analys?s.mp. [mp=title, abstract, original title, name of substance word, subject heading word, floating sub-heading word, keyword heading word, organism supplementary concept word, protocol supplementary concept word, rare disease supplementary concept word, unique identifier, synonyms] (6821)

26 structural equation model*.mp. [mp=title, abstract, original title, name of substance word, subject heading word, floating sub-heading word, keyword heading word, organism supplementary concept word, protocol supplementary concept word, rare disease supplementary concept word, unique identifier, synonyms] (17281)

27 20 or 21 or 22 or 23 or 24 or 25 or 26 (3114789)

28 19 and 27 (296)

29 limit 28 to english language (291)

Database: Embase Classic+Embase <1947 to 2020 Week 17>

Search Strategy:

--------------------------------------------------------------------------------

1 adhd.mp. or exp attention deficit disorder/ (63643)

2 attention deficit-hyperactivity disorder*.mp. (31845)

3 attention deficit hyperactivity disorder*.mp. (31845)

4 attention deficit disorder* with hyperactivity.mp. (1664)

5 addh.mp. (155)

6 1 or 2 or 3 or 4 or 5 (65796)

7 exp socioeconomics/ or socioeconomic*.mp. (453844)

8 exp income group/ or exp household income/ or exp personal income/ or exp family income/ or income.mp. or exp income/ (213889)

9 exp income group/ or exp household income/ or exp personal income/ or exp family income/ or income.mp. or exp income/ (213889)

10 poverty.mp. or exp poverty/ (57667)

11 social class.mp. or exp social class/ (38366)

12 economic status.mp. or exp economic status/ (150813)

13 educational status.mp. or exp educational status/ (77935)

14 parent*.mp. or exp parent/ (763580)

15 13 and 14 (11669)

16 7 or 8 or 9 or 10 or 11 or 12 or 15 (609340)

17 6 and 16 (2881)

18 (mediating or mediation or mediator* or mediated).mp. [mp=title, abstract, heading word, drug trade name, original title, device manufacturer, drug manufacturer, device trade name, keyword, floating subheading word, candidate term word] (1492361)

19 moderat*.mp. (828152)

20 attenuat*.mp. (448226)

21 indirect*.mp. (295941)

22 pathway*.mp. (1428970)

23 path analys?s.mp. (7761)

24 structural equation model*.mp. or exp structural equation modeling/ (19500)

25 18 or 19 or 20 or 21 or 22 or 23 or 24 (3931596)

26 17 and 25 (379)

27 limit 26 to english language (372)

Database: APA PsycInfo <1806 to April Week 4 2020>

Search Strategy:

--------------------------------------------------------------------------------

1 adhd.mp. or exp Attention Deficit Disorder with Hyperactivity/ (31259)

2 attention deficit-hyperactivity disorder*.mp. (27032)

3 attention deficit hyperactivity disorder*.mp. (27032)

4 attention deficit disorder* with hyperactivity.mp. (29380)

5 addh.mp. (133)

6 1 or 2 or 3 or 4 or 5 (37838)

7 exp Socioeconomic Class Attitudes/ or socioeconomic*.mp. or exp Family Socioeconomic Level/ or exp Socioeconomic Status/ (111702)

8 exp Poverty/ or poverty.mp. (29443)

9 exp Income Level/ or exp "Income (Economic)"/ or income.mp. (68026)

10 economic status.mp. (7780)

11 social class.mp. or exp Social Class/ (31191)

12 exp Educational Attainment Level/ or exp Educational Background/ or educational status.mp. (25231)

13 parent*.mp. (297648)

14 exp Parents/ (115763)

15 13 or 14 (321759)

16 12 and 15 (5181)

17 7 or 8 or 9 or 10 or 11 or 16 (185938)

18 6 and 17 (1500)

19 (mediating or mediation or mediator* or mediated).mp. [mp=title, abstract, heading word, table of contents, key concepts, original title, tests & measures, mesh] (178899)

20 moderat*.mp. (175026)

21 attenuat*.mp. [mp=title, abstract, heading word, table of contents, key concepts, original title, tests & measures, mesh] (42620)

22 indirect*.mp. (54270)

23 pathway*.mp. (92534)

24 path analys?s.mp. [mp=title, abstract, heading word, table of contents, key concepts, original title, tests & measures, mesh] (10215)

25 exp Structural Equation Modeling/ or structural equation model*.mp. (33143)

26 19 or 20 or 21 or 22 or 23 or 24 or 25 (498541)

27 18 and 26 (253)

28 limit 27 to english language (237)

**TOPIC:** (adhd or "attention deficit disorder* with hyperactivity" or "attention deficit hyperactivity disorder*" or "attention deficit hyperactivity disorder*" or addh) *AND* **TOPIC:** (socioeconomic* or poverty or income or "economic status" or "social class" or (("educational status") and (parent*))) *AND* **TOPIC:** (mediating or mediation or mediator* or mediated or moderat* or attenuat* or indirect* or pathway* or "path analys?s" or "structural equation model*")

*Indexes=SCI-EXPANDED, SSCI, A&HCI, CPCI-S, CPCI-SSH, ESCI Timespan=All years*

(230)
