# Supplementary material for: Factors that mediate the relationships between household socio-economic status and childhood Attention Deficit Hyperactivity Disorder (ADHD) in children and adolescents: A systematic review
Source: PLoS One. 2022 Mar 1;17(3):e0262988. doi: 10.1371/journal.pone.0262988 (PMC8887716; doi:10.1371/journal.pone.0262988)
Supplement: Attachment — Submitted filename: Comments.docx [file pone.0262988.s005.docx]

**Comments:**

**Minor issue is:**

1. Introduction: starting from line 58, you have wrote about the prevalence of ADHD in USA since 2011, your study time is 2020, why not discus (look) after 2011.
2. Results: starting line 166, you have screened n = 626 titles and abstracts for eligibility and n = 82 are assessed and 72 are exclude, what about the rest or the remaining titles and abstracts.
3. Line 168, you have assessed n = 82 titles and abstracts, and line 180 you have categorized by study type: Five studies were cohort studies, two were cross-sectional studies and one was a case-control study. When we add the type it is 8, how you categorized?
4. The figures are not visible.
5. Your article is a very lengthy (51 pages). It should be revised and shortened substantially.
6. Check your manuscript carefully with PLOS One author guidelines.
